# Grain number and grain yield distribution along the spike remain stable despite breeding for high yield in winter wheat

**DOI:** 10.1371/journal.pone.0205452

**Published:** 2018-10-10

**Authors:** Norman Philipp, Heiko Weichert, Utkarsh Bohra, Winfriede Weschke, Albert Wilhelm Schulthess, Hans Weber

**Affiliations:** 1 Department of Breeding Research, Leibniz Institute of Plant Genetics and Crop Plant Research, Corrensstr. 3, Gatersleben, Germany; 2 Department of Molecular Genetics, Leibniz Institute of Plant Genetics and Crop Plant Research, Corrensstr. 3, Gatersleben, Germany; Institute of Genetics and Developmental Biology Chinese Academy of Sciences, CHINA

## Abstract

Two winter wheat (*Triticum aestivum* L.) populations, i.e. 180 genetic resources and 210 elite varieties, were compared in a field trial to analyse how grain number and grain yield distribution along the spike changed during the breeding process and how this associates to yield-related traits. Elites showed in average 38% more yield compared to resources. This breeding improvement mainly derived from an increase in grains and yield per spike in addition to grains and yield per spikelet. These increments corresponded to 19, 23, 21 and 25%, respectively. Not much gain in thousand grain weight (4%) was observed in elites as compared to resources. The number of spikelets per spike was not, or even negatively, correlated with most traits, except of grains per spike, which suggests that this trait was not favoured during breeding. The grain number and grain yield distributions along the spike (GDAS and GYDAS) were measured and compared by using a novel mathematical tool. GDAS and GYDAS measure the deviation of a spike of interest from the architecture of a model spike with even grain and yield distribution along all spikelets, respectively. Both traits were positively correlated. Elites showed in average only a 1% improvement in GDAS and GYDAS values compared to resources. This comparison revealed that breeding increased grain number and yield uniformly along the spike without changing relative yield input of individual spikelets, thereby, maintaining the general spike architecture.

## Introduction

Wheat (*Triticum* spp.) accounts for 30% of global grain production and for 45% of cereal nutrition, thus representing a major food crop species [1]. In recent years the actual rate of wheat production increased by only 0.5% per year, which is much less than the required 1.4% that would be necessary to cope with a still growing human population [2, 3]. Therefore, improved wheat production must be achieved by further increasing the grain yield per area. However, the current increase of wheat yield decelerates and the harvest index approaches a theoretical limit. Moreover, the available genetic pool seems to be widely exhausted and modern breeding has further led to reduced genetic variability [1]. In this respect, exploiting wheat genetic resources could be promising to overcome this drawback [4].

Grain yield in wheat is predominantly sink-limited and grains grow under saturated source supply [5]. Increased assimilate partitioning to developing spikes and grains had the greatest impact on improving yield potential in wheat during the past, which increased the harvest index but with much less biomass gain [6]. The yield gain in the past mainly comes from an increased grain number per area rather than a higher grain size [7]. Grain yield is a complex trait and the result of components that interact in a multiplicative manner [8]. Several studies in wheat have detected some quantitative trait loci influencing grain yield that co-locate with those associated to its components, which suggests partially shared genetic control for these traits [9–12]. The yield components of wheat are multifaceted [13] and cover two main parameters: grain yield per area and grain yield per spike. Grain yield per area includes grains per spike, grain weight and spikes per area; whereas grain yield per spike comprises spikelet number per spike, grain number and grain size per spike and/or spikelet. There are multiple interactions and compensation mechanisms between the different yields components, dependent on genotype x environment x agronomy interactions [13]. In this sense, important yield-related traits such as grain weight and grain number are often negatively correlated [14].

The wheat spike contains a variable number of around 24 to 28 spikelets, each with several florets. Grains can differ in terms of developmental stage, weight, number and fruiting efficiency when compared among different spikelets and even within individual spikelets [15]. The middle spikelets have more and heavier grains than the basal and top spikelets [16]. Spikelet numbers, grain weight and grain numbers per spikelet have also a significant effect on thousand grain weight (TGW) and grain number per spike. The degree and rate of filling of the grains in individual spikelets varies highly by their position at the spike [17].

The natural variation represented in the wheat genetic resources, constitutes an important initiator of genetic advance [18]. Comparing yield-related traits between genetic resources and elite varieties can help to assess and understand the progress in breeding and selection. The objectives of this study were (i) to quantify the differences in grain yield and yield related traits between two populations of 180 genetic resources and 210 elite varieties of winter wheat (*Triticum aestivum* L.), (ii) to identify most relevant yield components responsible for grain yield improvement in elite varieties, and (iii), to examine in which manner grain number and grain yield distribution along the spike were changed during breeding.

## Material and methods

### Plant material

**Fig 1:**
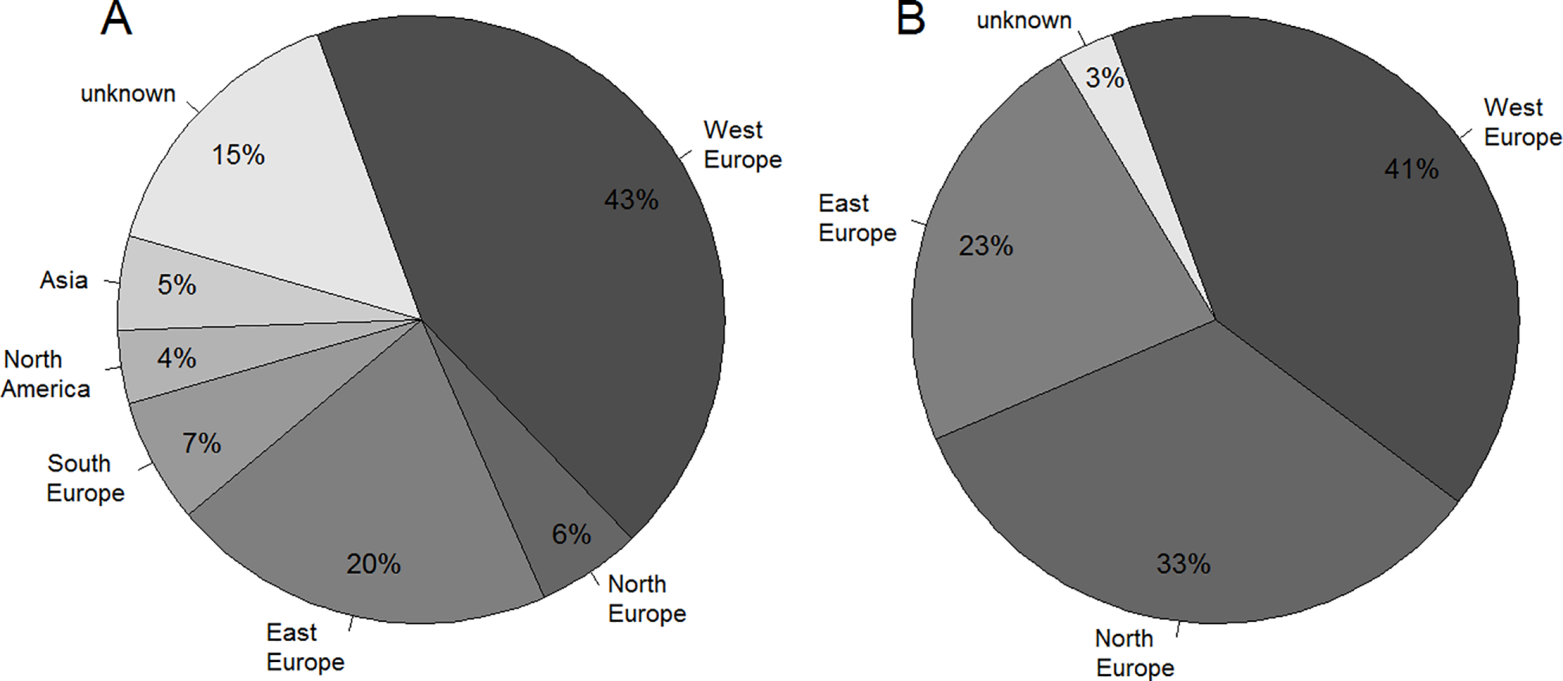
Origin of the two winter wheat populations. (A) Origin of 180 genetic resources and (B) origin of 210 elite varieties.

The present study includes two winter wheat populations. The first one comprehends 180 genetic resources randomly sampled from the German Federal *ex situ* Genebank of agricultural and horticultural crops maintained at the Leibniz Institute of Plant Genetics and Crop Plant Research Gatersleben. The second population is constituted by 210 European elite varieties derived from GABI-WHEAT [19] and VALID [20] projects (S1 Table). The majority of the accessions of the population of genetic resources originated from West Europe (43%), East Europe (20%), South Europe (7%) and North Europe (6%), while 5% and 4% came from Asia and North America, respectively. The origin of 15% of the accessions was unknown (Fig 1A). The varieties of the elite population derived from West Europe (41%), North Europe (33%) and East Europe (23%), while for the remaining 3% the origin was unknown (Fig 1B).

### Field trials

The populations were tested together using an incomplete block design (alpha-lattice) with two replications at Gatersleben, Germany (latitude 51° 49' 19.74" N, longitude 11° 17' 11.80" E, 110.5 m.a.s.l., black soil of clayey loam type, 9°C average annual temperature, 490 mm average annual rainfall). The variety Apache was repeated ten times within each replication in order to fill the number of plots per replication to 400 for optimal randomisation within incomplete blocks. The experimental unit corresponded to a plot of 5 m^2^ with a sowing density of 220 seeds per m^2^. Fertilizers, growth regulators, herbicides and fungicides were applied according to local agricultural practices. Before harvest, five main spikes were sampled from each plot in each replication and stored for further investigations. After threshing, grains harvested from each plot were weighed and grain yield was expressed in Mg ha^-1^.

### Investigation of spike architecture

In total, ~4,000 spikes (10 per genotype; 100 of the filler variety Apache) were investigated for number of spikelets per spike, number of grains per spikelet and grain yield per spikelet (g). Spikelets were carefully removed stepwise from bottom to the top. From each spikelet the grains were removed, cleaned and weighted. Grain yield per spike and the number of grains per spike as well as a measure for grain distribution along the spike (GDAS) and grain yield distribution along the spike (GYDAS) were calculated. The average trait value across the five investigated spikes per plot was used for further analysis. Grains from all five spikes of one plot were combined and analysed by MARVIN Seed Analyser (www.gta-sensorik.com) for TGW (g), grain length (mm), grain width (mm) and grain area (mm^2^).

The spikelets along a spike differ in their fertility. This can be measured by the grain number and the grain yield (g) of the individual spikelets. Hence, the total grain number and the total grain yield of a spike are distributed more or less even along the spike. In order to quantify which kind of distribution (even vs. uneven) was favoured by plant breeders`selection, we developed a measure to compare the evenness of GYDAS and GDAS among spikes. This measure is a mathematical term based on the cosine of the angle of two vectors and can be described in detail as the following:

A model spike was assumed with ideal GYDAS of absolutely even grain yield distribution along all spikelets and used for comparison with the spike of interest. Comparison of the spike of interest with the model spike occurs in an *n*-dimensional vector space, which dimensions are defined by the total spikelet number of the spike of interest. The geometrical difference in GYDAS between the two spikes is based on the scalar product of these two vectors:

$$cos∡(\vec{a},\vec{b})=\frac{\vec{a}∙\vec{b}}{\left|\vec{a}|∙|\vec{b}\right|},$$ (1)

where the directions of the two vectors differ from each other by a certain angle, reflecting the difference in grain yield distribution between the spike of interest *a* and a model spike *b*. The term is based on the difference in the cosine of angle between the model spike and the spike of interest. In addition to the number of spikelets per spike the term utilizes the parameters grain yield per spikelet and grain yield per spike to describe GYDAS or number of grains per spikelet and number of grains per spike to describe GDAS. For the example of GYDAS this can be written as:

$$GYDAS=\frac{\sum_{i=1}^{n}a_{i}{∙b}_{i}}{\sqrt{\sum_{i=1}^{n}a_{i}^{2}∙}\sqrt{\sum_{i=1}^{n}b_{i}^{2}}},$$ (2)

where *a*_*i*_ and *b*_*i*_ are the grain yield (or number of grains) of the *i*-th spikelet (among a total of *n* spikelets) of the spike of interest and the model spike, respectively. Due to the even grain yield distribution along all spikelets in the model spike, *b*_*i*_ is strictly equal to one and the last equation is reduced to:

$$GYDAS=\frac{\sum_{i=1}^{n}a_{i}}{\sqrt{\sum_{i=1}^{n}a_{i}^{2}}.\sqrt{n}}=\frac{\sum_{i=1}^{n}a_{i}}{\sqrt{n∙\sum_{i=1}^{n}a_{i}^{2}}},$$ (3)

where $\sum_{i=1}^{n}a_{i}$ is equal to the grain yield of the spike of interest (GYS):

$$GYDAS=\frac{GYS}{\sqrt{n∙\sum_{i=1}^{n}a_{i}^{2}}}.$$ (4)

**Fig 2:**
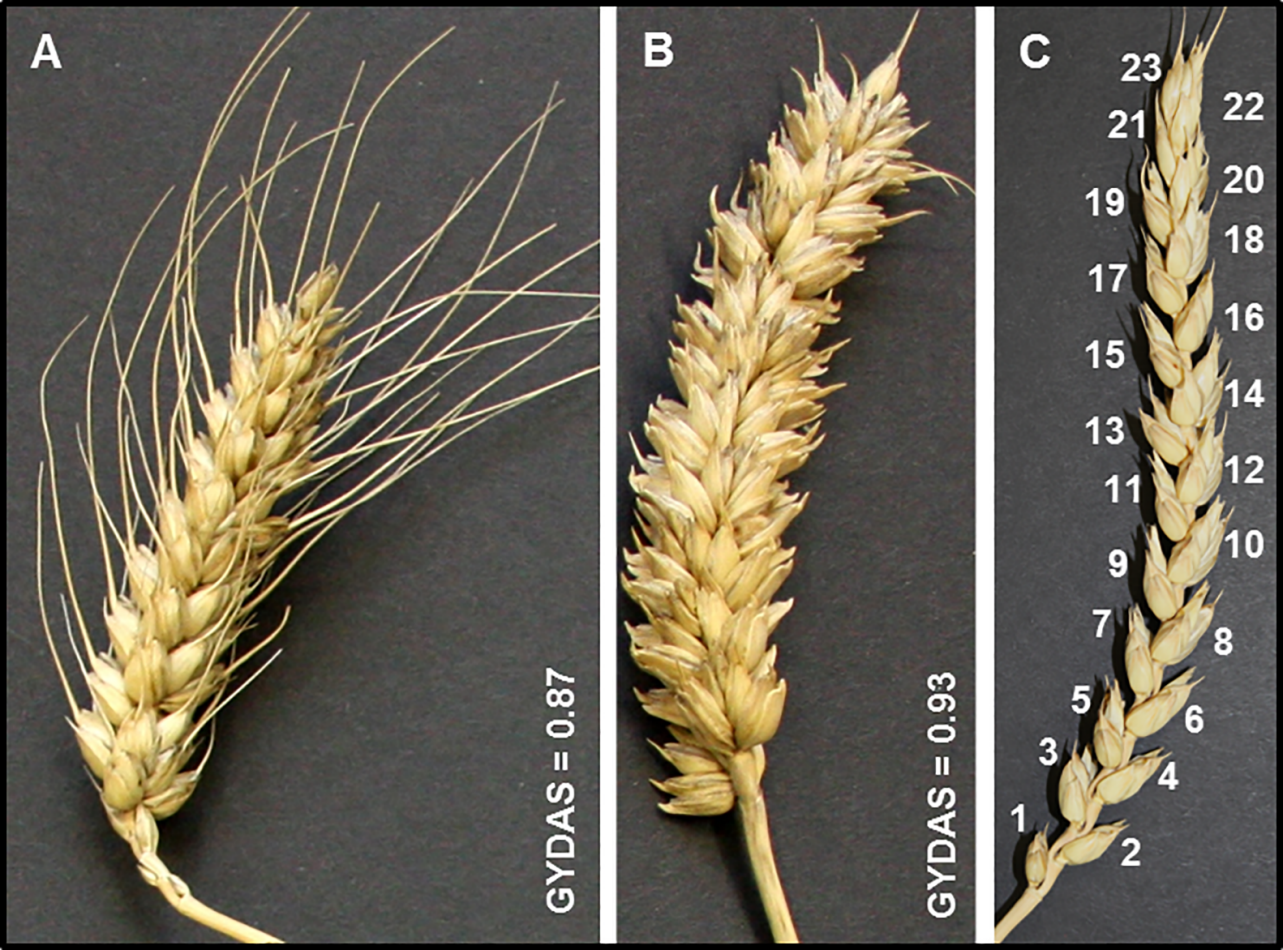
Contrasting values of the grain yield distribution along the spike (GYDAS). (A) The genetic resource TRI_1218 and (B) the elite variety Limerick as well as (C) an example spike showing spikelet numbering according to the position along the spike.

The cosine value is the measure of the distribution along the spike. The higher the cosine value, the better (more even) is GYDAS or GDAS (Fig 2). At a value of 1 the angle of difference is 0°, meaning the spike of interest has the same distribution as the model spike.

### Estimation of relevant quantitative genetic parameters

For analysing the phenotypic data we fitted the following linear mixed model for each trait:

$$y_{ijk}=\mu +g_{i}+r_{j}+b_{k(j)}+e_{ijk},$$ (5)

where y_*ijk*_ is the field performance of the *i*th genotype in the *j*th replication and *k*th block, μ the intercept, *g*_*i*_ the effect of the *i*th genotype, *r*_*j*_ the effect of *j*th replication, *b*_*k*(*j*)_ the effect of *k*th block nested within the *j*th replication, and *e*_*ijk*_, is the error of *y*_*ijk*_. For outlier correction the genotype was assumed as fixed factor, whereas replication and block were considered random. Records were removed as outliers if their standardized residuals exceeded a certain threshold according to [21]. Afterwards, the model in Eq (5) was fitted again in order to estimate best linear unbiased estimations (BLUEs). For estimation of variance components the outlier corrected data was fitted with the following modified model in order to separately estimate genetic variance components for the population of genetic resources and elite varieties:

$$y_{ijkl}=\mu +p_{l}+g_{i(l)}+r_{j}+b_{k(j)}+e_{ijkl},$$ (6)

where y_*ijkl*_ is the field performance of the *i*th genotype in the *j*th replication, *k*th block and *l*th population, μ the intercept, *p*_*l*_ the effect of the *l*th population, *g*_*i*(*l*)_ the effect of the *i*th genotype in the *l*th population, *r*_*j*_ the effect of *j*th replication, *b*_*k*(*j*)_ the effect of *k*th block nested within the *j*th replication, and *e*_*ijkl*_, is the error of *y*_*ijkl*_. Dummy variables [22] coded presence (1) of genotypes in *l*th population and absence (0) of genotypes belonging to the other population and were not shown in the model. For estimation of variance components of genotypes, replications blocks and errors, these were assumed as random factors, whereas the population mean was considered as fixed factor. The variance components were used to estimate the repeatability as:

$$Repeatability=\frac{\sigma_{G}^{2}}{\sigma_{G}^{2}+\frac{\sigma_{error}^{2}}{Nr.rep}},$$ (7)

where $\sigma_{G}^{2}$ refers to the genotypic variance, $\sigma_{error}^{2}$ to the error variance, and *Nr*.*rep* to the number replications. Significance of the difference in population means between genetic resources and elite varieties was tested by using a t-test for two samples comparison based on estimated BLUEs. The significance of genetic variance components was assessed by applying a likelihood ratio test which considered the respective likelihoods of full and reduced models. Genetic variances were considered significantly different between populations if their approximated confidence intervals (the genetic variances plus/minus twice their standard errors) did not overlap. Pearson’s correlations between the BLUEs of the traits were calculated separately for genetic resources and elite varieties.

In order to analyse the contribution of the individual spikelets to the overall grain number and grain yield of a spike in the populations of genetic resources and elite varieties we fitted the following model:

$$y_{ijkl}=\mu +({sp)}_{ij}+r_{k}+b_{l(k)}+e_{ijkl},$$ (8)

where *y*_*ijkl*_ is the grain number or grain yield of the *i*th spikelet (spindle step) in the *j*th population, *k*th replication and *l*th block, μ the intercept, (*sp*)_*ij*_ the effect of the *i*th spikelet (*s*) in the *j*th population (*p*), *r*_*k*_ the effect of the *k*th replication, *b*_*l*_ the effect of the *l*th block nested within the *k*th replication and *e*_*ijkl*_ the error of *y*_*ijkl*_. For estimation of BLUEs (*sp*)_*ij*_ was assumed as fixed factor all the others as random factors. Linear mixed models were implemented using ASReml-R [23]and all statistical procedures were executed in R environment [24].

## Results

### Genetic resources and elite varieties differ by an average grain yield disparity of 38.11%

**Fig 3:**
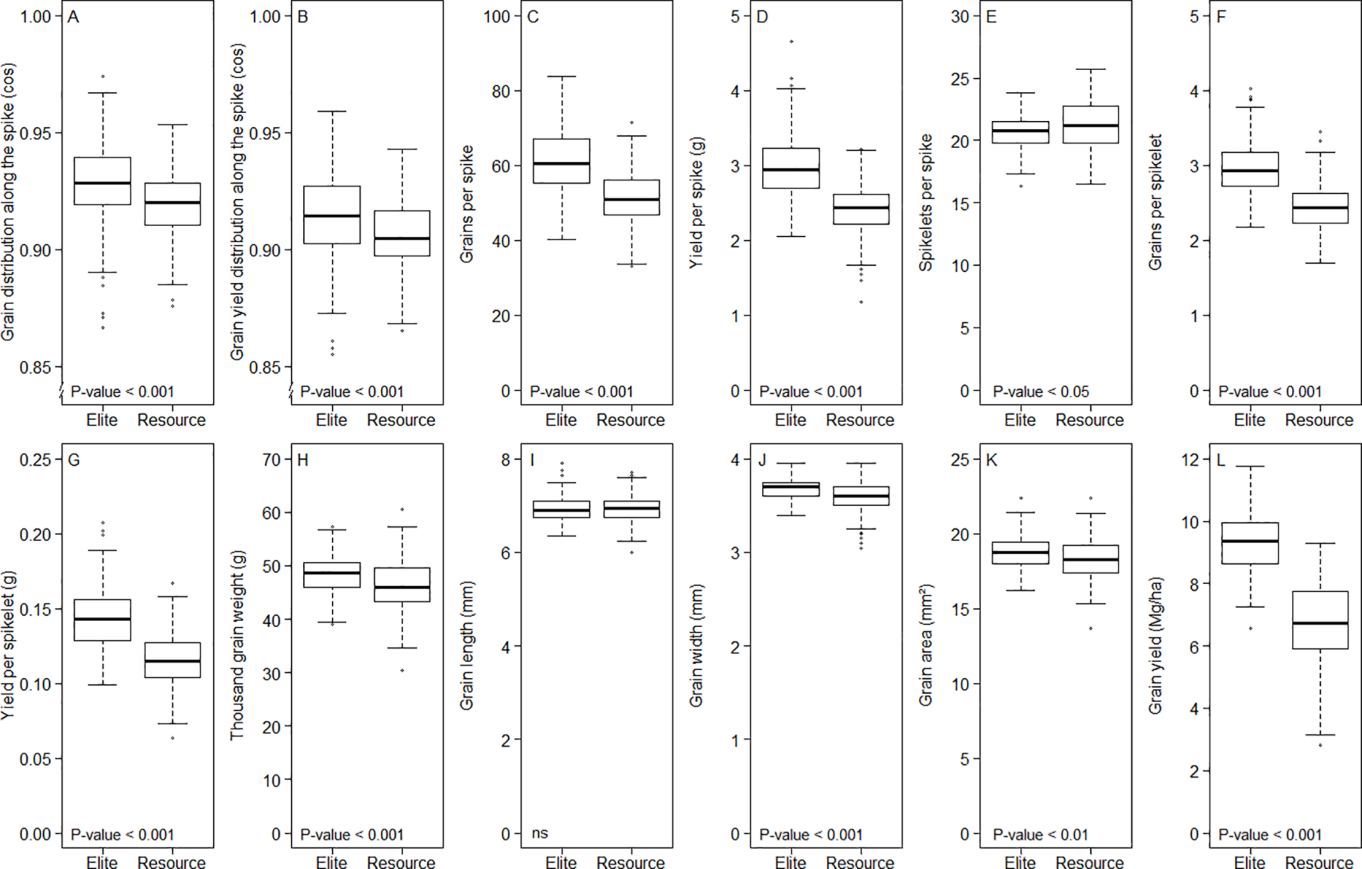
**Distribution of Best Linear Unbiased Estimates (BLUEs) of 12 yield-related traits (A-L), measured in two winter wheat populations of 180 genetic resources (Resource) and 210 elite varieties (Elite).** P-value indicates significantly different mean values between populations; ns, not significant.

Data quality, as indicated by repeatability estimates was moderate to high and varied between 0.46 for GDAS and 0.94 for grain length in the population of genetic resources and between 0.63 for GDAS and 0.93 for grain length in the population of elite varieties (Table 1). All genetic variance components were significantly different from zero (P-value < 0.001). Differences of the genetic variance between the populations were only significant (P-value < 0.05) for grain yield per spike, TGW, grain width, grain area and grain yield. While the genetic variance of grain yield per spike in the elite population was elevated by 109%, the genetic variances of the other mentioned traits were reduced by up to 59% compared to the population of the genetic resources. The differences in mean values between the two populations were significant (P-value < 0.05) for all investigated traits except for grain length. Minor increases in the means of the elite population compared to the genetic resources were observed for GDAS (1.06%), GYDAS (1.03%), grain area (2.21%), grain width (2.36%) and TGW (3.80%) (Table 1, Fig 3). Much higher increases were observed for grains per spike (19.05%) and grain yield per spike (23.22%) as well as grains per spikelet (21.25%) and grain yield per spikelet (25.03%), while the highest increase was found for plot grain yield (38.11%). The only trait with a minor reduction in mean value compared to the genetic resources was spikelets per spike (-1.95%).

**Table 1:** First and second degree statistics^a^ of 12 yield-related trait investigated in two winter wheat populations of 180 genetic resources and 210 elite varieties as well as differences of population means and variances^b^ in relation to the population of genetic resources.

Source	GDAS (cos)	GYDAS (cos)	Grains/ spike	Yield/ spike (g)	Spikelets/ spike	Grains/ spikelet	Yield/ spikelet (g)	TGW (g)	Grain length (mm)	Grain width (mm)	Grain area (mm^2^)	Grain yield (Mg/ha)
**Resources**												
***Min***	0.88	0.87	32.97	1.18	16.51	1.70	0.06	30.35	6.00	3.05	13.70	2.81
***Max***	0.95	0.94	71.31	3.21	25.68	3.45	0.17	60.54	7.70	3.95	22.40	9.28
***Mean***	0.92	0.91	51.27	2.40	21.08	2.43	0.11	46.43	6.94	3.60	18.33	6.72
$\sigma_{G}^{2}$	0.0001***	0.0001***	27.10***	0.06***	3.43***	0.05***	0.0002***	22.54***	0.08***	0.02***	1.66***	1.21***
$\sigma_{e}^{2}$	0.0002	0.0002	37.85	0.11	0.57	0.06	0.0002	4.98	0.01	0.01	0.36	0.60
***h***^**2**^	0.46	0.49	0.6	0.55	0.93	0.63	0.66	0.90	0.94	0.88	0.91	0.81
**Elites**												
***Min***	0.87	0.86	40.23	2.05	16.34	2.18	0.10	39.12	6.35	3.40	16.20	6.56
***Max***	0.97	0.96	83.71	4.66	23.85	4.02	0.21	57.27	7.90	3.95	22.40	11.75
***Mean***	0.93	0.91	61.03	2.96	20.66	2.95	0.14	48.20	6.94	3.68	18.74	9.28
$\sigma_{G}^{2}$	0.0002***	0.0002***	47.63***	0.13***	1.40***	0.09***	0.0003***	10.36***	0.07***	0.01***	0.86***	0.55***
$\sigma_{e}^{2}$	0.0002	0.0002	37.85	0.11	0.57	0.06	0.0002	4.98	0.01	0.01	0.36	0.60
***h***^**2**^	0.63	0.67	0.73	0.72	0.84	0.75	0.74	0.81	0.93	0.76	0.83	0.66
***Diff***_***Mean***_ ***(%)***	1.06***	1.03***	19.05***	23.22***	-1.95*	21.25***	25.03***	3.80***	0.07	2.36***	2.21**	38.11***
${Diff}_{\sigma_{G}^{2}}$ ***(%)***	94.04	109.99	75.75	108.77*	-59.02*	78.73	47.13	-54.04*	-14.96	-55.04*	-48.06*	-54.35*

^**a**^ *Min*, minimum; *Max*, maximum; *Mean*, average; $\sigma_{G}^{2}$, genetic variance; $\sigma_{e}^{2}$, error variance; *h*^2^, repeatability. Asterisks indicate if genetic variance is significantly different from zero.

^**b**^ *Diff*_*Mean*_, difference of population means in relation to the population of genetic resources; ${Diff}_{\sigma_{G}^{2}}^{2}$ difference of genetic variances in relation to the population of genetic resources. Asterisks indicate significant differences tested on absolute values based on t-test and approximated confidence intervals for *Diff*_*Mean*_ and ${Diff}_{\sigma_{G}^{2}}^{2}$, respectively.

* *P*-value < 0.05

** *P*-value < 0.01

*** *P*-value < 0.001

### Major contributions to grain number and grain yield per spike comes from the lower half of the spikelets

**Fig 4:**
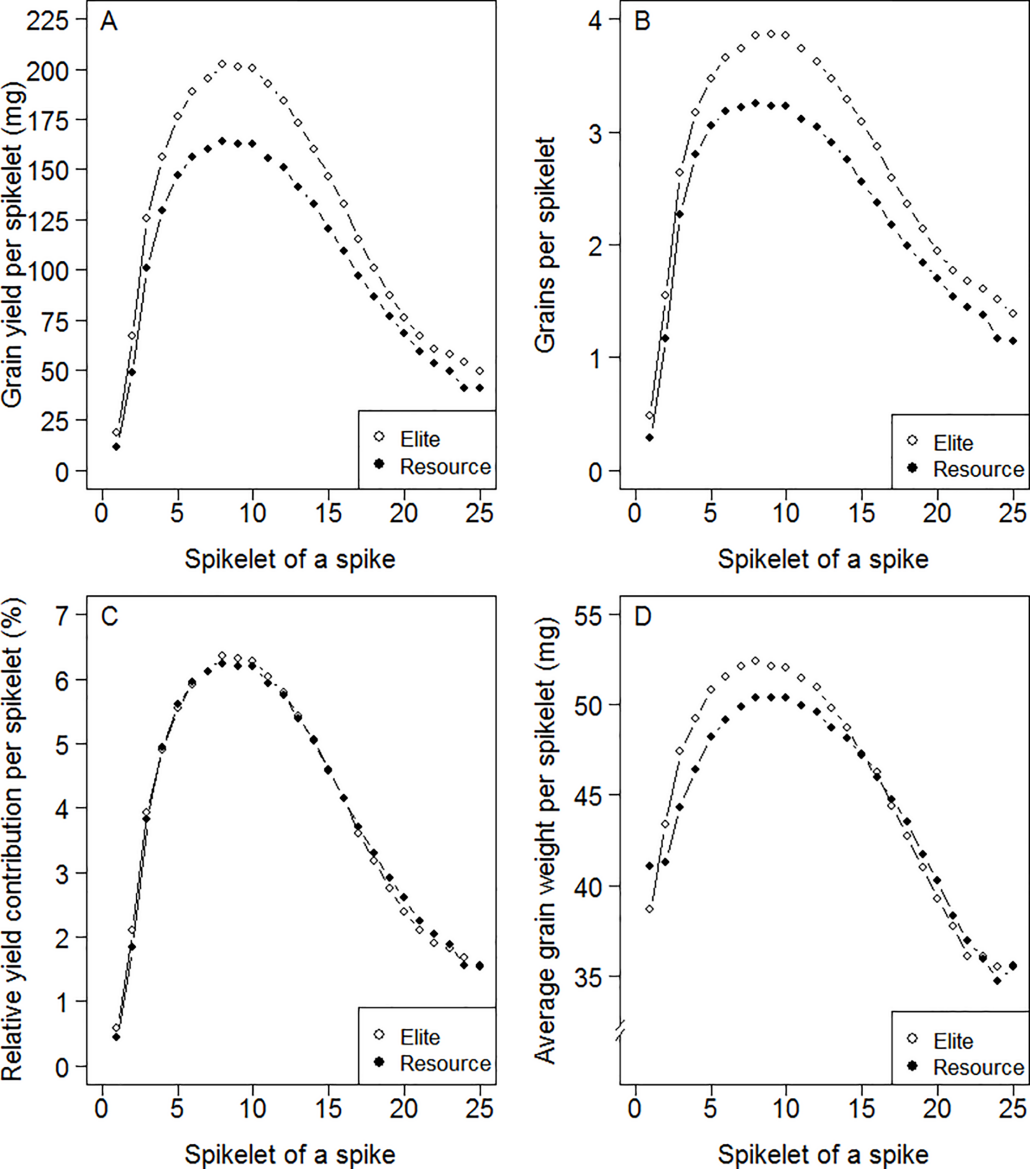
Distribution of yield-related traits along the spike displayed for the population of 180 genetic resources (Resource) and 210 elite varieties (Elite). (A) Grain yield per spikelet, (B) grains per spikelet, (C) relative yield contribution per spikelet and (D) average grain weight per spikelet.

By analysing the contribution of the individual spikelets to the overall grain yield and grain number of a spike (Eq 8) it was observed that grain yield in both elites and resources was unevenly distributed along the spikelets of the spike. Specifically, the contribution to grain yield was mainly concentrated in spikelets four to fourteen (Fig 4A). The same was true for the distribution of the number of grains along the spike (Fig 4B). The mean comparison showed that both number of grains and grain yield increased in the elites in all spikelets along the spike. However, the relative yield contributions per spikelet, i.e. the percentages of the contributions of the individual spikelets to the total spike yield, were practically identical between elites and resources (Fig 4C). Please note that GDAS and GYDAS are a measure to compare the evenness of grain number and grain yield distribution along the spike between the populations shown in Fig 4A and Fig 4B, respectively. The average grain weight of the individual spikelets, calculated by dividing the spikelet yield (Fig 4A) by the grains per spikelet (Fig 4B), was marginally increased for elites in the basal and middle part of the spike (Fig 4D). Taken together, the results indicate that the yield advantage of the elite spikes mainly comes from more grains along the entire spike, whereas the relative yield contributions of the individual spikelets along the spike were almost not altered.

### Correlations involving grain yield generally decreased in the population of elite varieties

Two groups of correlations were clearly identified within the trait correlation matrix (Table 2): Correlations whose magnitudes were conserved between populations and those whose magnitudes differed when shifting from resources to elite material (Table 2). Significant changes in arithmetic sign (+, -), i.e. when a positive significant correlation shifts towards a negative significant one by going from resources to elite material or vice versa, were not observed. We highlighted a few interesting examples portraying these findings in this section. In the population of elite varieties the highest, significant correlations (P-value < 0.001) were observed between GDAS and GYDAS (0.97), grain yield per spike and grains per spike (0.85), grains per spikelet and grains per spike (0.88), grains per spikelet and grain yield per spike (0.77), grain yield per spikelet and grain yield per spike (0.90) as well as grain yield per spikelet and grains per spikelet (0.83). The correlations among the grain dimension traits: TGW, grain length, grain width and grain area were in general high, with correlation coefficients up to 0.90 between grain area and TGW. In this respect, only the correlation between grain width and grain length was low (0.29). Similar correlations were also observed in the population of genetic resources. Interestingly, spikelets per spike tend to be negatively correlated to GDAS and GYDAS and positively correlated to grains per spike and grain yield per spike in both populations. Nevertheless, these correlations were on low to moderate levels. In the elite population GDAS was moderately correlated to grains per spike (0.51), grain yield per spike (0.51), grains per spikelet (0.69) and grain yield per spikelet (0.65). In contrast in the population of genetic resources GDAS was considerably less correlated to grains per spike (0.27), grain yield per spike (0.28), grains per spikelet (0.40) and grain yield per spikelet (0.37). A similar tendency was also observed for the correlations of the mentioned traits with GYDAS in both populations. There was no notable correlation observed for GDAS or GYDAS with grain yield for both populations, being the association between GDAS and grain yield of resources (0.18) only barely significant. In the population of genetic resources grain yield correlated moderately with grain yield per spike (0.39), grain yield per spikelet (0.43), grain width (0.41) and TGW (0.28), while in the elite population these correlations were approximately halved for grain yield per spike (0.26), grain yield per spikelet (0.20) and grain width (0.16). Last but not least, the correlation between grain yield and TGW was not significant in the elite population.

**Table 2:** Pearson correlation coefficients among 12 yield-related traits measured in two winter wheat populations of 180 genetic resources (upper triangle) and 210 elite varieties (lower triangle).

Trait	GDAS (cos)	GYDAS (cos)	Grains/ spike	Yield/ spike (g)	Spikelets/ spike	Grains/ spikelet	Yield/ spikelet (g)	TGW (g)	Grain length (mm)	Grain width (mm)	Grain area (mm^2^)	Grain yield (Mg/ha)
**GDAS (cos)**		0.95***	0.27***	0.28***	-0.151	0.40***	0.37***	0.09	-0.02	0.15*	0.10	0.18*
**GYDAS (cos)**	0.97***		0.15*	0.21**	-0.18*	0.29***	0.32***	0.14	0.04	0.17*	0.14	0.09
**Grains/ spike**	0.51***	0.51***		0.72***	0.46***	0.74***	0.39***	-0.22**	-0.28***	-0.01	-0.18*	0.23**
**Yield/ spike (g)**	0.51***	0.54***	0.85***		0.21**	0.62***	0.81***	0.46***	0.23**	0.55***	0.46***	0.39***
**Spikelets/ spike**	-0.24***	-0.26***	0.45***	0.33***		-0.25**	-0.39***	-0.33***	-0.21**	-0.16*	-0.25***	-0.13
**Grains/ spikelet**	0.69***	0.7***	0.88***	0.77***	-0.01		0.73***	-0.02	-0.16*	0.08	-0.03	0.33***
**Yield/ spikelet (g)**	0.65***	0.69***	0.69***	0.90***	-0.11	0.83***		0.63***	0.35***	0.62***	0.59***	0.43***
**TGW (g)**	0.07	0.14	-0.14*	0.35***	-0.20**	-0.06	0.46***		0.69***	0.87***	0.95***	0.28***
**Grain length (mm)**	0.04	0.08	-0.09	0.23**	-0.10	-0.06	0.27***	0.61***		0.41***	0.81***	-0.03
**Grain width (mm)**	0.19**	0.26***	0.10	0.50***	-0.04	0.14	0.54***	0.82***	0.29***		0.84***	0.41***
**Grain area (mm^2^)**	0.13	0.20**	-0.03	0.42***	-0.12	0.02	0.49***	0.90***	0.82***	0.75***		0.20**
**Grain yield (Mg/ha)**	0.10	0.13	0.22**	0.26***	0.17*	0.15*	0.20**	0.11	0.05	0.16*	0.12	

* P-value < 0.05

** P-value < 0.01

*** P-value < 0.001

## Discussion

Comparing yield-related traits between genetic resources and elite varieties can help to assess and understand breeding progress and selection. In this study, we focused on the development, description and validation of two new agronomic traits, GDAS and GYDAS, based on two populations of 180 genetic resources and 210 elite varieties of winter wheat. With the data collected in this context, we were further able to draw conclusions in which extent grain number and grain yield distributions along the spike were changed during the breeding process and how this is associated to other yield-related traits. Please consider that all found associations are limited to one environment and may modulate in other environments. Therefore, we were carefully with their interpretation and relied on supporting literature.

### Enhanced number of grains per spike explains approximately a half of yield improvement in elites

Approximately a half of the 38% yield improvement achieved by breeding in elites was associated with a concomitant 23% increment in grain yield per spike (Table 1, Fig 3). This increment in grain yield per spike was in turn associated to a concomitant increase in grain numbers per spike (19% increment), grain number (21%) and grain yield per spikelet (25%). The other half of the yield improvement observed in elites may be mainly attributed to an increased spike number per m^2^, since this trait, along with the grain yield per spike, corresponds to one of the main grain yield components in wheat. Moreover, it has been frequently reported that there is a closer relationship between grain yield and the number of grains than between grain yield and grain weight [7, 25–28]. For instance, modern varieties of English winter wheat have 59% more grains due to 30% extra grains per spike and 14% more ears per m^2^ but with similar TGW [29], while the comparison of old and modern durum wheat varieties in Italy and Spain revealed that breeding increased grain numbers per spike by 23% due to a higher grain numbers per spikelet [30]. The observation that the grain yield improvement in elites was accompanied by an increased number in grains per spike but without much gain in TGW (3.8% increment) in our study confirms these past findings. While breeding history reveals that genetic improvement is associated with more grains per area, elite varieties seem not to produce more total biomass, indicating that the source to sink ratio has been decreased [31]. Thus, grain yield improvement comes from increased biomass partitioned to the grains. Most important was the development of semi-dwarf lines by introducing the *Rht* alleles, which altered assimilate partitioning to the ears and increased spike fertility [28]. Another key feature contributing to enhance grain yield was the increment in water soluble carbohydrates in stems and leaves at anthesis. This enlarges the source reserves and therefore the available carbon to be allocated to filling grains [7]. These key improvements shifted the negative relationship between TGW and grain numbers per area in the elites, which fill more grains without much loss in TGW [28].

### Enhancement of grains per spike is obtained by promoting individual spikelet fertility rather than increasing spikelets per spike

Since enhanced grain numbers per spike is associated with higher yield, increasing spikelet number per spike would potentially boost grain numbers per spike. However, spikelets numbers per spike decreased in the elites by 2% and therefore has not been a favoured selection target. Even though the number of spikelets per spike is moderately correlated with the number of grains per spike in elites (+0.45) and resources (+0.46), correlations with grain yield and other yield related traits are poor or even negative (Table 2). This indicates that more spikelets per spike are rather disadvantageous for grain yield. Accordingly, for Australian wheat, the number of spikelets is related to grain number but not grain yield [32, 33]. Spikelet development in winter wheat is dependent on vernalization and can be increased by extending growing period, plant spacing, temperature, nitrogen nutrition and light intensity [34, 35]. This demonstrates that assimilate competition within the developing spike plays a role [36]. The low association with grain yield and the fact that spikelet numbers are related to a longer growing period [32] and spike developmental phase [37] could explain why increasing spikelets has not been favourably selected.

### Breeding altered the average values of many yield-related traits in elite varieties

The selection of plant material for high yield and TGW may have also indirectly altered related traits (Tables 1, 2, Fig 3). This so-called correlated response to selection is a function of the correlation among traits and trait heritabilities [38, 39]. Co-selection may have influenced average trait performances between the two populations and their genetic variances. For instance, selection for higher TGW significantly increased the mean and reduced the genetic variance of TGW, but also positively increased the correlated traits grain width and grain area. The effects of trait co-selection are not always obvious. One notable exception is co-selection of grain yield and its trait component yield per spike. Even though breeders prioritised to select plant material with high grain yield and thereby co-selecting higher yield per spike; the genetic variance of grain yield decreased (-54.35%) but that of the yield per spike concomitantly increased in the elites (108.77%). One plausible explanation for this is that insufficient yield per spike can be compensated by increased number of spikes per m^2^ in plant material with multi-culm growth habit [8, 40]. In this sense, the selection of high yielding material may have admitted selection of genotypes that not necessarily have high yield per spike, which, in turn, increased the genetic variance for yield per spike in the elites. Since the spike number per m^2^ was not assessed here, we cannot exclude other causes. Last but not least, several trait correlations changed in magnitude, i.e. they became weaker or stronger, when comparing resources with elites (Table 2). Nonetheless, these shifts could simply be artificial due to trait values truncation [41], and mass selection by breeders.

### Breeding has not altered the uneven grain number and grain yield distributions along the spike

During most of the grain filling period wheat is rather sink- than source-limited [42]. Alleviating sink limitation was recently favoured to further improve grain yield [43]. Analysing spike architecture in both elites and resources shows that grain number, grain yield and average grain weight per spikelet differ dependent on the position of the spikelet in the spike (Fig 4A–4D). In both elites and resources, grain yield distributions of the spikelets are very similar, with spikelet four to fourteen accounting for 64% of yield per spike. This is encouraging if it is considered that these spikelets located in the lower spike half correspond to approximately 44% of the total number of spikelets. In this sense, improving the spike architecture towards more yield in the upper spike parts could be promising to overcome sink limitations.

GDAS and GYDAS as measures of the evenness of grain number and grain yield distribution along the spike, respectively, were very similar and highly correlated in both elites and resources (Fig 3A–3B). Both, GDAS and GYDAS are only slightly improved by 1% in the elites indicating that a stable spike architecture is maintained (Fig 3A and 3B; Table 1). Thus, the percentage and contribution of individual spikelets to the total spike yield remains nearly unchanged for both elites and resources (Fig 4C). In other words, breeding improved the grain number and yield uniformly along the spike without changing the relative yield input of individual spikelets. Such limited alteration suggests that these traits were not targeted by breeding probably because of low correlation to grain yield (Table 2). Alternatively, maintenance of stable grain and grain yield distributions could reflect that characteristic features of spike physiology and development are not easy to alter by breeding with simultaneous gain of yield. Since grains in spikes and spikelets develop asynchronously and degree and rate of filling varies highly by spike position, distal grains remain smaller than basal grains, due to later filling and slower initial filling rate, and also by synchronous maturation among different grains [44, 45]. The slower filling rate of distal grains is associated with lower abscisic acid concentrations and higher concentrations of ethylene and 1-aminocyclopropane-1-carboxylic acid [17]. Competition for assimilates within the spike was reported by decreasing assimilate availability by low irradiance, which reduced TGW in upper compared to basal grains, whereas high temperature reduces grain size in all positions [42]. Possible limitations of transport capacities and competition for assimilates between spikelets and/or florets could impact yield [2]. A potential issue could be resistance to assimilate movement within spikes and/or spikelets [46]. Disparity in dimensions of vascular bundles in different spike segments could be critical affecting ultimate size and grain numbers along the rachis [47]. Eventually, genetic yield gain was not accompanied by similar increases in the vasculature size of the spike. Accordingly, no clear association was found between genetic improvement and magnitude of vascular system in peduncles of the spike [48].

### Thousand grain weight has been a secondary breeding target

While breeding progress in the elites is largely achieved by enhanced grain number per spikelet and grains per spike, TGW has only been marginally improved, (Table 2) and grain length was not altered (Fig 3H and 3I). TGW is highly correlated with grain width, area and grain yield per spikelet and spike but only moderately with grain length in both elites and resources (Table 2), [14]. Regulation of grain length and width is largely independent. Length is determined very early in grain development and driven by pericarp elongation [49, 50]. Width is determined later at grain filling and determined by endosperm cell division, driving force for sucrose between vasculature and endosperm and storage activity [51, 52]. Increased TGW was apparently achieved by improving mainly grain width (2.36%) and area (2.21%) rather than length (Table 1). Multiple yield components and spike characteristics are associated with the *Q* gene, present in all modern wheat varieties. *Q* is associated with reduced ratios of grain length to weight, leading to shorter and rounder grains [53]. TGW and grain width provide an index for milling quality. Larger and shorter grains improve flour extraction rate and end use quality [16, 54, 55]. Therefore, TGW was mainly selected as quality parameter rather than as trait for grain yield improvement. Furthermore, average grain weight per spikelet is increased only in the lower and middle spike, from spikelet one to thirteen (Fig 4D). A trade-off has often been reported between grain number and grain weight in wheat, ascribed to non-competitive reasons [54, 56]. When grain number and yield were increased, the proportion of smaller grains in distal spike positions also augmented, thus lowering the average grain weight [57]. However, such view cannot be confirmed here because the gain in grains and yield in the elites is proportionally distributed over the whole spike and not preferentially higher in distal regions (Fig 4C). The presence of smaller distal grains can be rather assigned to limitation and/or competition of assimilate supply at the whole spike level since grain number can be modified by assimilates allocated to the spike [58]. The ectopic expression of a sucrose transporter in the wheat endosperm increased individual grain weight but decreased grain number per spike [59, 60]. Thus, these genotypes could suffer from competition between grains for assimilates and therefore from potential constraints in the supply of the spike. Different features might control assimilate supply such as loading and unloading within the vascular system and short distance transport within spike, rachis and spikelets [61].

### Further validation of GDAS and GYDAS is necessary to prove the practical use for plant breeding

Since the number of grains per spikelet is correlated to the spikelet yield (0.73 for genetic resources and 0.83 for elite varieties) GDAS and GYDAS are quite similar. Thus, both traits could be measured interchangeable in practice. Although breeding practically did not alter GDAS and GYDAS, this does not rule out the use of these traits for indirect selection of grain yield. To assess the ability of GDAS and GYDAS as indirect traits for selection of grain yield, an experiment should be performed in which enough variation on these three traits is available within a base population and the plant material is purely selected based on either the indirect traits or grain yield itself. The comparison of the genetic gains attained by both procedures will reveal the advantages of indirect selection using GDAS and GYDAS over direct selection for grain yield.

## Conclusion

Breeding essentially increased grain numbers per area in the elites by enhancing grains per spike and spikelets without much gain in TGW. More spikelets per spike were obviously not preferably selected because it may increase grains per spike but not yield and with potential compensations in TGW and/or grains per spikelet. While sink limitation was alleviated by allocating more assimilates to spikes, the uneven yield contribution of the individual spikelets remained stable. Limited success to improve spike architecture suggests that either this trait was not targeted by breeding or its maintenance reflects characteristic features of spike physiology and development, which are not easy to alter with concomitant gain in yield. Manipulating the uneven grain number or grain yield distribution of spikelets along the spike could generate yield benefits possibly by addressing assimilate loading, unloading within the vascular system and short distance transport within spike, rachis and spikelets. These features control and constrain transport capacities and competition between spikelets and grains.

## Supporting information

S1 Table Passport and registration information of investigated genetic resources and elite varieties. (XLSX)

S2 Table Raw data of the spikes as well as the derived data sets analysed by the Eqs 5 and 8 and their corresponding Best Linear Unbiased Estimates (BLUES). (XLSX)
